# Potential therapeutic role of pyroptosis mediated by the NLRP3 inflammasome in type 2 diabetes and its complications

**DOI:** 10.3389/fendo.2022.986565

**Published:** 2022-10-27

**Authors:** Xiang Li, Gui-Ying Xiao, Tao Guo, Yu-Jie Song, Qiu-Mei Li

**Affiliations:** Department of Endocrinology and Metabolic Diseases, Dalian University Affiliated Xinhua Hospital, Dalian, Liaoning, China

**Keywords:** type 2 diabetes, pyroptosis, NLRP3 inflammasome, complications, therapy

## Abstract

As a new way of programmed cell death, pyroptosis plays a vital role in many diseases. In recent years, the relationship between pyroptosis and type 2 diabetes (T2D) has received increasing attention. Although the current treatment options for T2D are abundant, the occurrence and development of T2D appear to continue, and the poor prognosis and high mortality of patients with T2D remain a considerable burden in the global health system. Numerous studies have shown that pyroptosis mediated by the NLRP3 inflammasome can affect the progression of T2D and its complications; targeting the NLRP3 inflammasome has potential therapeutic effects. In this review, we described the molecular mechanism of pyroptosis more comprehensively, discussed the most updated progress of pyroptosis mediated by NLRP3 inflammasome in T2D and its complications, and listed some drugs and agents with potential anti-pyroptosis effects. Based on the available evidence, exploring more mechanisms of the NLRP3 inflammasome pathway may bring more options and benefits for preventing and treating T2D and drug development.

## Introduction

T2D is a multifactorial autoimmune disease characterized by glucose and lipid metabolism disturbances, insulin resistance (IR), and absolute or relative insulin deficiency (1, 2). In addition, chronic inflammation runs through the entire process of the development of diabetes and its complications (3–5), exacerbating metabolic imbalances and the development of complications (6, 7). Worldwide, the incidence of T2D is still increasing (8, 9), and its complications remain a significant cause of death (10). Inflammatory response and metabolic disorders are a vicious cycle in type 2 diabetes, and chronic inflammation, blood sugar, and lipid disorders promote each other (11–13). A better understanding of the inflammatory response may have a significant effect on the treatment and outcome of T2D.

**Figure 1:**
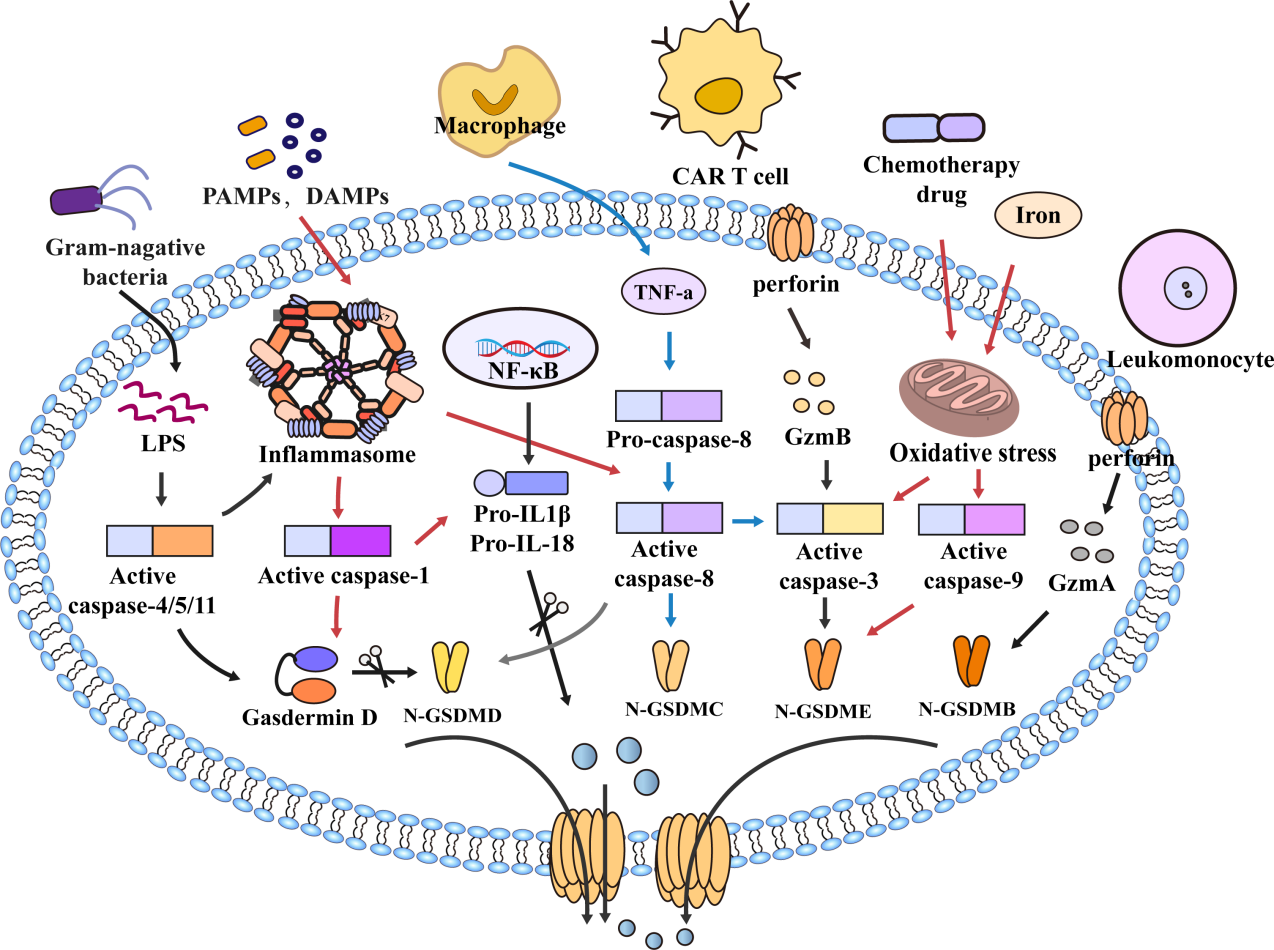
Molecular mechanism of pyroptosis. In the caspase-1-dependent pathway, PAMPs and DAMPs mediate inflammasome assembly and activate caspase-1, which cleaves GSDMD and pro-IL-1β/18. N-GSDMD forms non-selective pores on the cell membrane surface, and IL-1β and IL-18 are secreted out of the cell membrane through the pores formed by N-GSDMD, which further leads to cell lysis and death. In the caspase-1-independent pathway, LPS of bacteria activates caspase-4/5/11 and cleaves GSDMD to trigger pyroptosis. However, caspase-4/5/11 can also activate inflammasome assembly and caspase-1 to induce pyroptosis. In addition, in the caspase-8-mediated pathway, TNF-α induces the activation of caspase-8, cleavage of GSDMD leads to pyroptosis, and caspase-8 can also cleave GSDMC and GSDME, Furthermore, the NLRP3 inflammasome also activate Caspase-8. Granzyme also mediates pyroptosis, such as GzmB released from CAR T cells induces pyroptosis by activating caspase-3 and cleaving GSDME. In the caspase-9-mediated pathway, chemotherapy drugs and iron cause mitochondrial stress, activate caspase-3/9, and cleave GSDMB/E to mediate pyroptosis. Furthermore, GzmA in cytotoxic leukomonocyte enters target cells *via* perforin and cleaves GSDMB resulting in cell pyroptotic death. Similarly, GSDMB/C/E also forms pores to mediate the secretion of inflammatory mediators out of the membrane, amplify the inflammatory response, and promote cell death.

As a form of programmed cell death, pyroptosis is essential in maintaining physiology homeostasis and pathogen invasion (14). With the discovery of gasdermins family, the scope of pyroptosis has expanded. The earliest study of pyroptosis dates back to 1986, Friedlander’s study of mouse macrophage death and content release (15). It was not until 2001 that D’Souza et al. coined the term pyroptosis to describe inflammatory programmed cell death (16). In 2015, pyroptosis was defined as the inflammatory programmed cell death mediated by gasdermins (17). The gasdermins family includes gasdermin A/B/C/D (GSDMA/B/C/D), gasdermin E (GSDME, also known as DFNA5), and DFNB59 (Pejvakin, PJVK) (18). When pyroptosis occurs, gasdermins are cleaved by caspases into two fragments (the N-terminal pore-forming domain (PFD) and the C-terminal repressor domain (RD)); the N-terminal PFD oligomerizes and forms pores in the cell membrane, leading to the release of inflammatory factors and caspases, which promote cell pyroptotic death (18–20). For a long time, pyroptosis was thought to be caspase-1-induced monocyte death, and now the definition of pyroptosis has been expanded to include caspase-4/5/11 (21). Current research suggests that caspase-1 and caspase-4/5/11 are only related to pyroptosis. In contrast, caspase-2, caspase-7, and caspase-10 are only associated with apoptosis (22–26). Other caspases such as caspase-3/8/9 are involved in the process of pyroptosis and apoptosis (17, 27–32), and play an essential role in the occurrence and development of innate immune diseases, autoimmune diseases and tumors (33–37). Previous studies suggested that Caspase-3 was an executor of apoptosis, but a recent study found that Caspase-3 can induce pyroptosis by cleaving GSDME (27, 38). Apoptosis-related protein caspase-8 can also directly cleave GSDMC and GSDMD to induce pyroptosis (28, 39, 40). In addition, caspase-9 is also involved in pyroptosis by activating caspase-3 (41) **(** **Figure 1** **)**. With the deepening of research, the mechanism of pyroptosis has gradually become apparent, and the relationship between pyroptosis and disease has also been more explored and studied.

Recent studies have shown that pyroptosis, especially the NLRP3 inflammasome-mediated pyroptosis, plays a vital role in the progression of diabetes and its complications (42). However, the relationship between diabetes and pyroptosis has not been fully understood, and the proportion of progression in diabetes is not known. Although some articles on similar topics have been published in recent years (42), in this review, we will mainly discuss the current progress of pyroptosis and summarize the connection between pyroptosis mediated by NLRP3 and T2D and its complications more comprehensively. In addition, we will highlight some current therapeutic strategies targeting the NLRP3 inflammasome signaling pathway, which may provide new targets for treating diabetes.

## Molecular mechanism of pyroptosis

### Canonical pyroptosis

The classical pyroptotic pathway mediates caspase-1 activation through inflammasome assembly, triggering the cleavage of GSDMD and the release of inflammatory substances such as IL-1β and IL-18 into the extracellular space, leading to further expansion of the inflammatory response (43–45). The inflammasome is a complex of multiple molecules that begins to assemble through recognizing danger signals by cytosolic pattern recognition receptors (PRRs) (46). PRRs, also known as inflammasome sensors, include NLRP1, NLRP3, NLRC4, AIM2, and pyrin (47, 48). Most inflammasomes are composed of three functional domains: NOD-like receptors (NLRs) of inflammatory sensors, the apoptosis-associated speck-like protein containing a caspase recruitment domain (CARD) (ASC) and caspase (49–51). Whether the N-terminus of NLRs contains CARD or Pyrin domain (PYD), NLRs are divided into NLRPs or NRCs. The N-terminus of NLRCs has one or more CARD domains, such as NLRC4; the N-terminus of NLRPs is PYD, such as NLRP1 (containing PYD and CARD domains) and NLRP3 (52–56). Unlike NLRs, ASCs have both PYD and CARD domains (53). The inflammatory sensors AIM2 and pyrin are also involved in inflammasome assembly despite lacking NLRs. AIM2 consists of a C-terminal HIN-200 domain and an N-terminal PYD fragment (57–60). However, pyrin comprises an N-terminal PYD, two B-boxes, a coiled part, and a C-terminal B30.2 domain (also known as SPRY/PRY domain). Mouse Pyrin lacks the C-terminal B30.2 domain but is functionally similar to humans (53, 61, 62).

**Figure 2:**
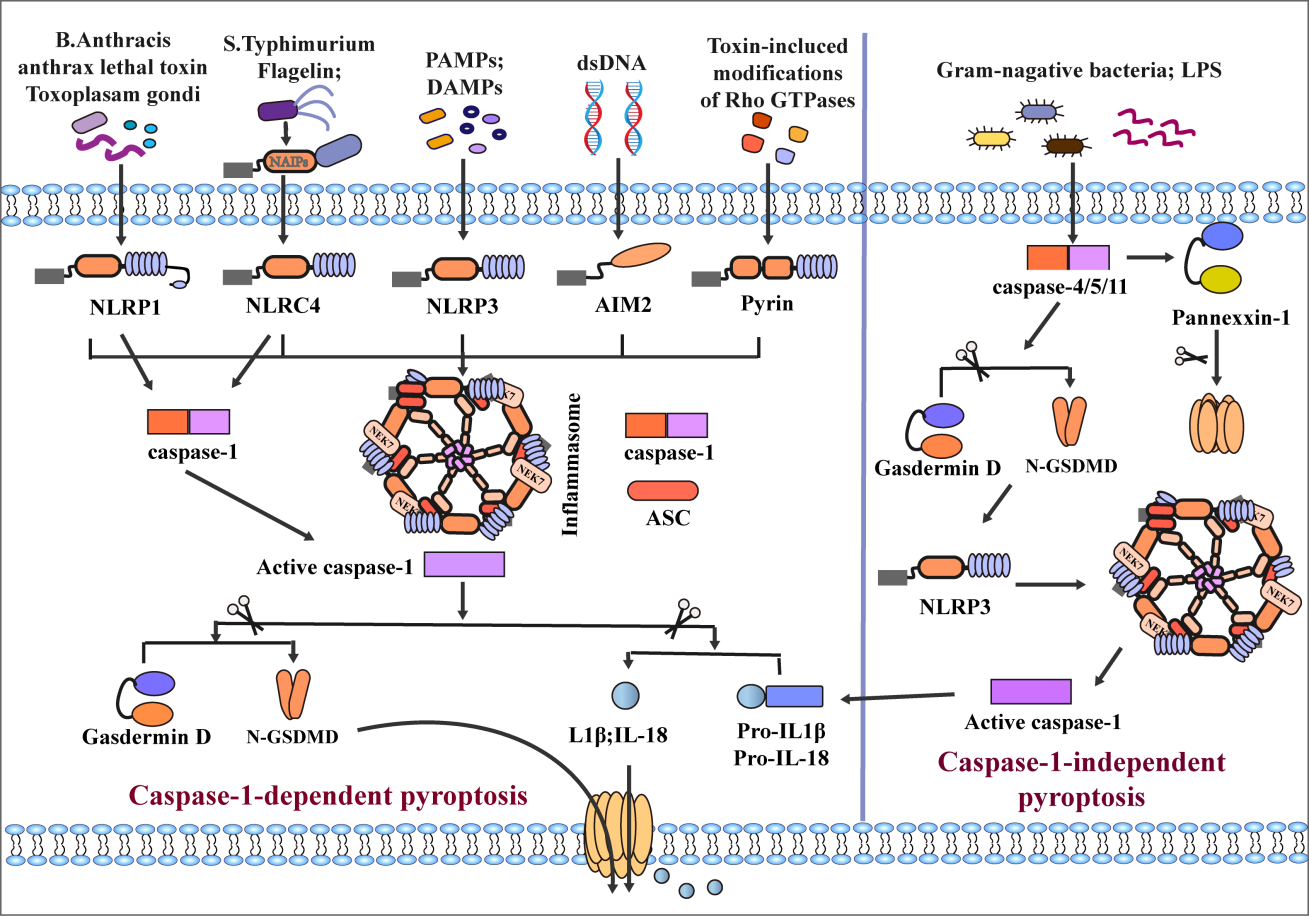
Classical and non-classical inflammasome assembly. The canonical pathway is mediated by caspase-1. When NLRP1, NLRC4, NLRP3, AIM, and Pyrin inflammatory sensors receive different stimuli, recruiting ASC and caspase-1 to mediate the assembly of inflammasomes and activate caspase-1. Among them, the assembly of NLRP3 and NLRC4 inflammasomes requires the participation of NEK7 and NAIPs, respectively, and NLRP1 and NLRC4 can directly activate case page-1 due to their CARD domains. After activation of Caspase-1, GSDMD and pro-IL-1β/18 is cleaved, and the pore formed by GSDMD on the cell membrane surface mediates secretion of IL-1β/18 out of the cell and induces pyroptosis. The non-canonical pathway is mediated by caspase-4/5/11; LPS of Gram-negative bacteria directly induces caspase-4/5/11 activation and cleaves GSDMD and Pannexin-1. N-GSDMD induces caspase-1 activation by activating NLRP3 inflammasome assembly, which mediates the release of inflammatory mediators and pyroptosis.

Many microbial infections, as well as non-microbial diseases, can cause inflammasome activation, for example, Val-boroPro (Talabostat, PT-100) (63) Toxoplasma gondii (55), Bacillus anthracis and its anthrax lethal toxin (64, 65) can cause the activation of NLRP1. NLRP3 can recognize pathogen-associated molecular patterns (PAMPs) and danger-associated molecular patterns (DAMPs), infectious microbes or their evolutionarily conserved molecular patterns are called PAMPs. After the body is infected, an inflammatory response is induced to cause damage. DAMPs are immunostimulatory molecular patterns in sterile inflammation and are positively correlated with damage. DAMPs are released when tissue is damaged and initiate an inflammatory response (66). Such as advanced glycation end products (AGEs), high mobility protein (HMGB-1), S100 protein family, heat shock protein family (HSPs), toxins, extracellular matrix (ECM), nucleic acids and ATP (67–70), induce inflammasome activation with the involvement of the kinase NEK7 (71–73). NLRC4 is activated by the binding of NAIP proteins and ligands (flagellin and type 3 secretion system (T3SS) proteins) (74–76). AIM2 is activated by combining the C-terminal HIN-200 domain with cytoplasmic dsDNA (57–60, 77). Another inflammatory sensor, pyrin, is induced to activate and assemble through small GTPases of the Rho family (61, 78, 79). Rho-inactivating toxins (Clostridium difficile glycosyltransferase TcdB, Vibrio parahaemolyticus VopS) and the Yersinia pestis GTPase-activating protein (YopE) and cysteine protease (YopT) can also induce pyrin activation (80, 81). Activating these sensors results in oligomerization, which forms an inflammasome complex by recruiting ASC and caspase1. Interestingly, the inflammatory sensors NLRP1 and NLRC4, due to the CARD domain at the N-terminus, do not require the recruitment of ASCs to activate caspase-1 directly (53). Interestingly, inflammatory sensors NLRP1 and NLRC4, due to their N-terminal containing CARD domains, can directly activate caspase-1 without recruiting ASC. After inflammasome assembly, caspase-1 is hydrolyzed into two fragments (82); On the one hand, activated caspase-1 hydrolyzes GSDMD into a C-terminus of 22 kDa (C-GSDMD) and an N-terminus of 31 kDa (N-GSDMD). N-GSDMD can form pores in cell membranes (83, 84). On the other hand, caspase-1 cleaves pro-IL-1β/18 into mature IL1β and IL-18, which are released extracellularly through pores formed by GSDMD, resulting in cell pyroptotic death (17–19, 85, 86) **(** **Figure 2** **)**.

### Non-canonical pyroptosis

In the caspase-1-independent pyroptosis pathway, human caspase-4/5 or mouse caspase-11 can be directly activated by N-terminal CARD combined with lipopolysaccharide (LPS) (87–89). After activation of caspase-4/5/11, GSDMD is cleaved into C/N-GSDMD, N-GSDMD is transferred to the cell membrane to form plasma membrane pores, and the assembly of NLRP3 inflammasome is initiated at the same time (90). However, in the caspase-1-independent pathway, many studies suggested that caspase-4/5/11 cannot directly cleave pro-IL-1β/18, which requires activation of the NLRP3/caspase-1 pathway through N-GSDMD to induce induction Maturation and secretion of IL-1β/IL-18 (31, 91–93). Some studies have also found that caspase-4 can directly cleave pro-IL-1β and pro-IL-18 (94). In addition, Yang et al. found that caspase-11 can specifically cleave Pannexin-1, causing the release of ATP (95). Meanwhile, cleavage of GSDMD by caspase-4/5/11 leads to the efflux of K^+^ and IL-1β/IL-18, eventually leading to pyroptosis (17, 92) **(** **Figure 2** **)**. It is worth noting that Guanylate-binding proteins (GBPs) play an important role in non-canonical pyroptosis. GBPs could protect against bacterial infection, when cells are infected with LPS, GBPs would contribute to LPS release into the cytosol and activation of the noncanonical caspase-11 inflammasome, GBPs contribute to secretion of IL-1βand IL-18, and induction of pyroptosis (96, 97).

### Other signaling pathways

Traditionally, caspase-3 mainly promotes the occurrence of apoptosis. However, recent studies have found that activation of caspase-3 can cleave GSDME into N/C-terminal fragments. Similar to N-GSDMD, N-GSDME can cause the formation of pores in the cell membrane and promote cell pyroptotic death (38, 98). In addition, Orning and Sarhan et al. found that Yersinia induced activation of caspase-8 after infection of mouse macrophages, resulting in the lysis of GSDMD (28, 29). Wu Qiao et al. found that metabolite α-KG can mediate pyroptosis of Hela cells *via* caspase-8/GSDMC (99). Hou et al. also proposed that PD-L1 mediates caspase-8/GSDMC activation in breast cancer cells, resulting in pyroptotic death of breast cancer cells (39); Jiang et al. reported that caspase-1, 3, and 7 could mediate the cleavage of GSDME leading to pyroptosis (100). Furthermore, Chemotherapeutic drugs, iron also mediate the activation of the caspase-9/GSDME/caspase-3 axis to induce pyroptosis. Interestingly, recent studies have found that the Granzyme family can cause pyroptosis (100). Zhang Z et al. found that GzmB can directly cleave GSDME to induce pyroptosis and inhibit tumor growth (101). Similarly, Liu Y et al. found that chimeric antigen receptor (CAR) T cells can activate GSDME to induce pyroptosis (102). Zhou Z et al. reported that GzmA derived by leukomonocyte induces pyroptosis by cleaving GSDMB at Lys229/Lys244 sites (103) **(** **Figure 2** **)**.

## NLRP3 inflammasome-mediated pyroptosis and type 2 diabetes

NOD-like receptor (NLR) family pyrin domain-containing 3 (NLRP3) is an important PRR in the cytoplasm. The NLRP3 inflammasome consists of a sensor (NLRP3), an aptamer (ASC), and an effector (caspase 1). The NLRP3 inflammatory sensor is composed of a C-terminal leucine-rich repeat (LRR), a nucleotide-binding and oligomerization domain (NACHT), and an N-terminal PYD domain that recruits caspases (104). Activation of the NLRP3 inflammasome involves two signals, the first signal is the initiation of NLRP3 gene transcription mediated by the NF-κB pathway, and the second signal is the activation of NLRP3 inflammasome sensors (105). The NLRP3 sensor self-oligomerizes through homotypic NACHT domain interactions. Oligomeric NLRP3 recruits ASCs through homotypic PYD-PYD domain interactions and induces ASC aggregation into a macromolecular focal point called the ASC speck. Subsequently, the assembled ASCs recruit proaspase-1 through homotypic CARD–CARD domain interactions to form the NLRP3-ASC-caspase-1 protein complex which is known as the NLRP3 inflammasome (106).

In type 2 diabetes, A variety of metabolites and factors such as glucose and fatty acids (107), LPS released by the gut microbiota (108, 109), mitochondrial reactive oxygen species (mROS) (110), the international association for preventive pediatrics(IAPP) (111), ceramide (112), amino acid homocysteine (113) and ATP (114) can activate the NLRP3 inflammasome. After stimulation, the NLRP3 inflammasome is activated with the participation of the ligand NIMA-related kinase 7 (NEK7) (71–73).

**Figure 3:**
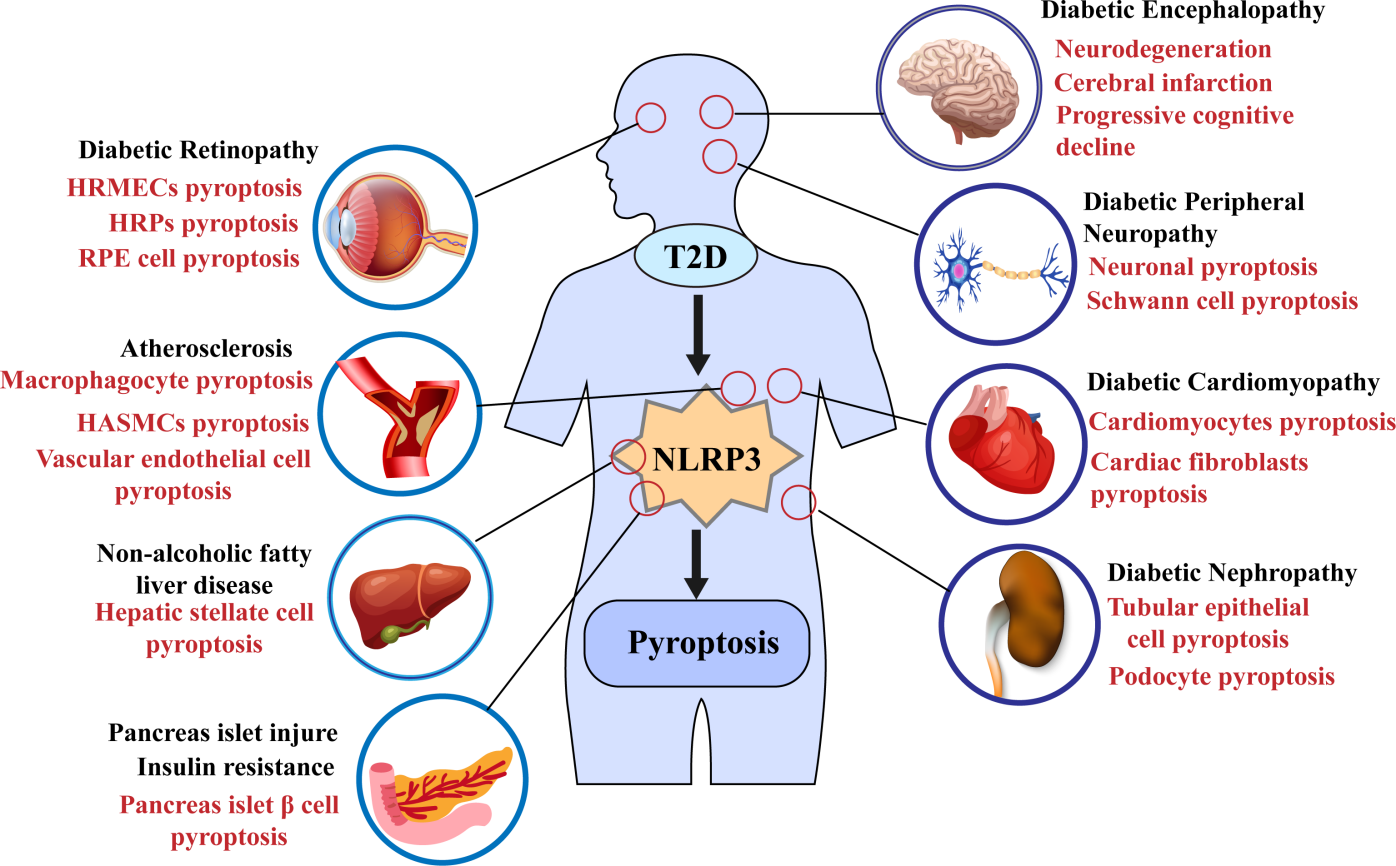
Pyroptosis mediated by the NLRP3 inflammasome in type 2 diabetes and its complications.

In 2010, Tschopp et al. first proposed that the NLRP3 inflammasome may be involved in the progression of T2DM (115). Subsequent studies have shown that NLRP3 inflammasome activation can aggravate IR and lead to further damage to islet β cells and promote T2D progression (5, 112, 116–119); some recent studies have also demonstrated that the activation of the NLRP3 inflammasome mediates the pyroptosis of pancreatic β cells (108, 120); for example, in the study of Yuan, J et al., the use of the NLRP3 inhibitor MCC950 ameliorated islet cell damage (121). Furthermore, pyroptosis mediated by the NLPR3 inflammasome plays a vital role in the progression of various complications such as diabetic nephropathy (DN) and diabetic cardiomyopathy (DCM) (**Figure 3**) (122–125). Recent studies have shown the potential therapeutic role of the NLRP3 inflammasome/pyroptosis signaling pathway in type 2 diabetes and its complications.

## Microvascular complications

### Diabetic nephropathy

As one of the microvascular complications of diabetes, DN is considered a sterile inflammatory disease and the leading cause of death in end-stage renal disease and patients with type 2 diabetes (126). Pyroptosis mediated by the NLRP3 inflammasome plays an important role in the development of diabetic nephropathy (122, 127, 128). In a previous study, Shahzad K et al. found that activation of the NLRP3 inflammasome in non-myeloid-derived cells exacerbates DN (127). A recent study showed that the expression levels of pyroptosis-related proteins such as NLRP3, caspase-1, and IL-1β were significantly increased in STZ-treated diabetic rats (129). An X et al. also found that in the HFD/STZ diabetic mouse model, the expression levels of pyroptosis-related proteins, including NLRP3 inflammasome, caspase-1, and GSDMD, were increased (130). Chen J et al. also found the same result, NLRP3 inflammasome recognizes risk signals, activates GSDMD, and inflammatory substances such as caspase-1 and IL-1β trigger pyroptosis and induce an inflammatory response. And it was confirmed by the TUNEL experiment that pyroptosis promotes the progress of DN (131). In addition, other studies have demonstrated that pyroptosis mediated by the NLRP3 inflammasome is associated with inflammation and fibrosis in DN, leading to aggravation of DN, the phenomenon was improved after the use of the caspase-1 inhibitor VX-765 (132–134). Notably, in renal tubular epithelial cells (TECs) and podocytes, NLRP3 inflammasome-mediated pyroptosis is critical in promoting DN progression (135–137).

### Podocyte pyroptosis

Podocytes are highly differentiated atypical epithelial cells of the kidney that are non-dividing, unique insulin-sensitive cells in the glomerulus. Participate in forming the filtration barrier and maintain the normal filtration function of the glomerulus (138). Podocyte loss is a central factor in DN proteinuria (139). Many studies have found podocyte pyroptosis closely related to diabetic nephropathy (140). HG inhibited the survival of podocytes in a dose-dependent manner and increased the levels of pyroptosis-related proteins such as ROS, IL-1β, and IL-18 in cells (141). Notably, activation of the NLRP3 inflammasome in podocytes promotes glomerular inflammation and glomerulosclerosis progression (142). In addition, D-ribose promotes NLRP3 inflammasome activation in type 2 diabetes to induce podocyte injury and glomerulosclerosis, the caspase-1 inhibitor YvAD significantly blocks podocyte injury (143). ABAIS et al. demonstrated that in a diabetes model, the protein expression levels of caspase-11 and GSDMD-N in podocytes were increased, and the expression of podocyte markers nephrin and podocin were decreased, podocyte loss and foot process fusion, promoting the expression of inflammatory factors such as NF-κB, IL-1β and IL-18 (140). Recent studies have found that the mammalian target of rapamycin (mTOR) regulates inflammation by binding to NLRP3 (144–146). Wang T et al. show that mTOR and NF-κB inhibitors reduce renal podocyte injury (147). In addition to the mTOR/NLRP3 signaling pathway, miRNAs can mediate the activation of the NLRP3 inflammasome and trigger podocyte pyroptosis under high glucose conditions. For example, Ding et al. demonstrated that miR-21-5p induces podocyte pyroptosis (148). In the study of Zhan et al., Long Non-Coding RNA (lncRNA) NEAT1 promoted podocyte pyroptosis by regulating miR-34c and regulated the expression of NLRP3, Caspase-1, and IL-1β in a mouse model of diabetes (129). Thioredoxin-interacting protein (TXNIP)/reactive oxygen species (ROS)/NLRP3 pathway is also involved in mediating podocyte pyroptosis and promoting the progression of DN (135). These findings above highlight the role of podocyte pyroptosis induced by the NLRP3/Caspase-1/IL-1β axis in diabetic nephropathy. Some of the latest cell studies also inhibit podocyte pyroptosis by interfering NLRP3 inflammasome signaling pathway, such as using the NLRP3 inflammasome inhibitor MCC950, which is expected to be used in treating diabetic nephropathy (135, 149–151).

Tubular epithelial cell pyroptosis

TECs are responsible for renal reabsorption and are closely related to the deterioration of renal function. The damage of TECs is also a critical link in DKD (152). Under HG conditions, TECs are more susceptible to glucose and lipid metabolism disorders, inflammatory responses, and hemodynamic changes, and the high-glucose environment produces ROS and releases various inflammatory factors, resulting in renal interstitial inflammation and fibrosis (122). Zhang et al. found that Caspase-11-mediated pyroptosis of TECs plays an essential role in acute kidney injury (137). In addition, lncRNAs are closely related to the progression of DN; for example, Xie et al. found that lncRNA GAS5 regulates the NLRP3 inflammasome-caspase-1 axis to mediate the pyroptosis of TECs (153). Similarly, Zhu et al. confirmed that the expression of NLRP3, caspase-1, IL-1β, p-IL-1β and GSDMD-N was up-regulated in HG-induced human tubular cells (HK-2), and the pyroptosis of HK-2 is associated with lncRNA KCNQ1OT1 (154). In the study of Wang et al., HG activated TLR4/NF-κB signaling pathway to mediate the pyroptosis of GSDMD-related TECs, and TLR4 inhibitor TAK-242 significantly ameliorated the damage of TECs (155). Interestingly, TXINP/NLRP3 pathway is also involved in mediating TECs pyroptosis; Ke et al. demonstrated that HG activates TXINP/NLRP3 pathway to mediate NRK-52E cell pyroptosis and kidney injury (134). HG also promotes TXNIP/NLRP3/caspase-1 pathway activation to mediate HK-2 pyroptosis (156).

### Diabetic retinopathy

As a common microvascular complication in diabetic patients, diabetic retinopathy (DR) is one of the leading causes of visual impairment in adults worldwide. Activation of the NLRP3 inflammasome may be involved in the pathogenesis of DR (157). For example, in the study by Yu et al., caspase-1 and GSDMD were activated after induction using advanced glycation end-products-modified bovine serum albumin (AGE-BSA), promoting inflammatory factors IL-1β, IL-18, and LDH release (158). Loukovaara et al. found elevated levels of pyroptosis-related proteins in DR (159). After induction with STZ, the expression levels of NF-κB, NLRP3, and caspase-1 were up-regulated in DR (160). In addition, HG can induce pyroptosis of human retinal microvascular endothelial cells (HRMECs) and human retinal pericytes (HRPs) (161, 162). NLRP3 inflammasome promotes cone cell death in a P23H rhodopsin retinal degeneration model (163). Za et al. also demonstrated HG-induced retinal pigment epithelium (RPE) cell pyroptosis (164). In recent years, more and more studies have been conducted on the mechanism of DR pyroptosis. RNA, methyltransferase-like protein 3 (METTL3), P2X7 purinergic receptor (P2X7R)/NLRP3, and ROS/TXNIP/NLRP3 pathways may all be related to DR pyroptosis (158, 164, 165). Targeted intervention in the activation of NLRP3 inflammasome may be beneficial for the prevention and treatment of DR.

## Macrovascular and Cardiac Complications

### Atherosclerosis

The risk of macrovascular disease is significantly increased in diabetes. Atherosclerosis (AS) in the aorta, coronary artery, and cerebral basilar artery is one of the complications of diabetes and the leading cause of cardiovascular events in patients with diabetes (166). A growing number of studies have demonstrated that many risk factors for type 2 diabetes, such as high glucose and high fat, can activate the NLRP3 inflammasome in endothelial cells (ECs) and macrophages to mediate pyroptosis and exacerbate the progression of AS (167). NLRP3 inflammasome and pyroptosis in atherosclerotic plaques are positively correlated with plaque rupture and vascular inflammation (168). In the study by An et al., carotid artery injury in T2D model rats was associated with elevated levels of NLRP3, caspase-1, and IL-1β (169). Rat aortic AS was associated with tissue activation of the NLRP3 inflammasome and NF-κB signaling in the Zucker diabetic fat (ZDF) model rat (170). Song et al. confirmed that HG promotes NLRP3 inflammasome activation and IL-1β secretion in ECs (171). Furthermore, in the study of Chen et al., HG increased the expression levels of NLRP3, caspase-1, and IL-1β in human arterial smooth muscle cells (HASMCs) (172). NLRP3 inflammasome, caspase-1, IL-1β, and IL-18 expression were upregulated in aortic ECs and vascular smooth muscle cells (VSMCs) in HFD-fed mice (173). The above studies further confirmed that pyroptosis mediated by the NLRP3 inflammasome promotes the progression of AS in a diabetes model, and targeting the pyroptosis associated with the NLRP3 inflammasome may have a potential therapeutic effect on type 2 diabetes with AS.

### Diabetic cardiomyopathy

DCM is a prevalent CVD, characterized by systolic dysfunction and left ventricular hypertrophy. Many risk factors, such as hyperglycemia, insulin resistance, increased oxidative stress, mitochondrial dysfunction, cardiomyocyte damage, and endothelial dysfunction, contribute to the development of DCM (174). A previous study found that the hearts overexpressed NLRP3, caspase-1, and IL-1 in diabetic rats (175). Pyroptosis associated with the NLRP3 inflammasome plays an important role in the pathogenesis of DCM (176). Silencing the NLRP3 gene improves cardiac inflammation, pyroptosis, and myocardial function (177). In cardiac tissue, pyroptosis occurs mainly in cardiomyocytes (CMs) and cardiac fibroblasts (CFs) (178–180). The death of CMs and CFs leads to cardiac remodeling and left ventricular dysfunction.

### Cardiomyocyte pyroptosis in DCM

Cardiomyocytes maintain the systolic and diastolic functions of the heart and ensure the blood supply of the whole body (123). Under high glucose conditions, the NLRP3 inflammasome was activated through multiple pathways to induce pyroptosis in CMs. For example, hyperglycemia promoted cardiomyocyte pyroptosis by activating the AMPK-TXNIP/NLRP3 signaling pathway (134, 181), and pyroptosis-associated TLR4 and NLRP3 inflammasome expression was increased in HG-treated H9C2 cardiomyocytes (182). HG also induced cardiomyocyte H9c2 pyroptosis by activating the NF-kB/NLRP3 pathway (177). ROS/NLRP3 and JNK/NLRP3 signaling pathways also promoted cardiomyocyte pyroptosis (183, 184). In addition, ELAV-like protein 1 (ELAVL1), LncRNA Kcnq1ot1, and miRNA were all involved in regulating cardiomyocyte pyroptosis in DCM (185–187). The death of CMs promotes DCM progression, leading to ventricular remodeling and even heart failure.

### Cardiac fibroblasts Pyroptosis in DCM

Cardiac fibrosis is one of the main pathological features of DCM, and CFs injury plays an essential role in this process. CFs are considered semi-occupational inflammatory cells that play an immunomodulatory role in the heart. Pyroptosis mediated by the NLRP3 inflammasome as pro-inflammatory programmed cell death is closely related to the damage of CFs (188). The NLRP3 inflammasome was activated with the participation of LncRNA, ROS, miRNA, and other mediators in the HG state, leading to cardiac fibroblast pyroptosis, promoting collagen synthesis and aggravating cardiac tissue fibrosis (189, 190). Some recent studies have further confirmed that CFs pyroptosis plays an important role in the pathogenesis of DCM (191, 192). It is necessary to explore further the mechanism of CFs pyroptosis for the prevention and treatment of DCM.

## Diabetic neuropathy

Extensive research evidence suggests that pyroptosis is also an important regulatory mechanism for diabetic neuropathy (125). Pyroptosis can lead to neuronal death and exacerbates diabetic neuropathy processes such as ischemic stroke, cognitive impairment, spinal cord injury (SCI), and peripheral neuropathy (193). In addition, pyroptosis mediated by the NLRP3 inflammasome also occurs in the optic and enteric nerves under high glucose conditions (194, 195).

### Diabetic encephalopathy

In recent years, pyroptosis in diabetic encephalopathy has received more and more attention. T2D induces pyroptosis by inducing activation of the NLRP3 inflammasome, aggravating neurodegeneration, cerebral infarction, and progressive cognitive decline (125). For example, Hong et al. found that the NLRP3 inhibitor MCC950 ameliorated cerebral ischemia/reperfusion (I/R) injury and reduced ischemic stroke risk in diabetic mice (196). In Wang et al., microglia pyroptosis exacerbates I/R injury, and NLRP3-specific inhibitor MCC950 ameliorated cerebral I/R injury in diabetic mice (197); Li et al. also found that hippocampal neurons in STZ-induced diabetes model mice mediated pyroptosis through the NLRP3 signaling pathway, accompanied by mouse depression-like behaviors (198). Similarly, Che et al. found that neuronal expression levels of NLRP3 inflammasome and pyroptosis were increased (199). Pyroptosis mediated by the NLRP3 inflammasome is a complex process in the progression of DE, and both lncRNAs and miRNAs play important roles in the process of pyroptosis (197, 199). Further exploring the mechanism of pyroptosis, especially the NLRP3 inflammasome signaling pathway, may be an essential strategy for the therapy of DE.

### Diabetic peripheral neuropathy

As one of the common complications of diabetes, diabetic peripheral neuropathy (DPN) is characterized by chronic inflammation, axonal degeneration, loss of unmyelinated fibers, and irreversible neuronal damage (165). Excessive ROS production leading to NLRP3 inflammasome activation promotes the level of pyroptosis in DPN (200). In Cheng et al.’s study, HG-induced the activation of NF-κB and NLRP3 inflammasome mediated pyroptosis in Schwann cells and promoted DPN progression (201). Similarly, Li et al. demonstrated that high glucose exacerbates demyelination in a mouse model of T2DM (202). Furthermore, in the study by Xu et al., high fat promoted the activation of the TXNIP/NLRP3 inflammasome in a diabetic mouse model (203). At the same time, miRNAs were also involved in the process of neuronal pyroptosis in diabetic mice (199). These findings proved that pyroptosis related to NLRP3 plays an important role in the development of DPN.

## Other complications

Pyroptosis mediated by the NLRP3 inflammasome also promoted the progression of diabetes-associated nonalcoholic fatty liver disease (NAFLD) syndrome. HG and saturated fatty acids acted together in the liver to exacerbate inflammation and endoplasmic reticulum stress to destabilize mitochondria (204), and long-term inflammatory response led to hepatocyte pyroptosis and promoted liver fibrosis (205). Activation of inflammasome directly stimulated hepatic stellate cells (HSCs) to increase the secretion of matrix metalloproteinases (MMPs) and accelerate the process of liver fibrosis (206). In addition, pyroptosis of stellate cells mediated by NLRP3 inflammasome further promotes liver fibrosis (207). After a specific knockout of the NLRP3 gene in hepatocytes, liver inflammation was significantly reduced (208). These studies suggested that pyroptosis mediated by NLRP3 inflammasome is vital for preventing and treating NAFLD.

## Potential drugs and agents that inhibit NLRP3 inflammasome signaling pathways

In recent years, there have been more and more studies on the mechanism of T2D pyroptosis, and many clinical drugs and compounds have demonstrated specific anti-pyroptosis effects (**Table 1**). For example, metformin, a first-line drug for diabetes treatment, inhibited the activation of NLRP3 inflammasome and cardiomyocyte pyroptosis (209–211), reducing myocardial ischemia-reperfusion injury (210). GLP-1 receptor agonists liraglutide and exenatide reduced neurological damage and inhibited NAFLD and cardiomyocyte pyroptosis in diabetic rats by inhibiting NLRP3 inflammasome activation (212–215); Similarly, exendin-4 also inhibited the activation of NLRP3 under HG conditions and alleviated cardiomyocyte pyroptosis (181). Furthermore, SGLT-2 inhibitor dapagliflozin and DPP4 inhibitor saxagliptin reduced NLRP3 inflammasome activation and delayed the progression of DCM in diabetic mice (216).

**Table 1:** Potential drugs and agents that inhibit NLRP3 inflammasome signaling pathways.

Drugs	Complications	Natural or Angents	Complications
Metformin	DCM	Melatonin	DCM, DPN, AS
Liralutide	DCM, DPN, NAFLD	H3 relaxin	DCM, DR
Exenatide	NAFLD, DCM	Exendin-4	DCM
Dapagliflozin	DCM	Nab	DN
Saxagliptin	DCM	miRNA	DN, DCM, DR, DPN
Chinese medicine	DN, DCM, DPN, DR	LncRNA	DN, DCM, DPN
…		MCC950	DR
		…	

Various active substances in traditional Chinese medicine also improved the pyroptosis of diabetes and its complications by inhibiting the NLRP3 inflammasome pathway, such as ginsenoside Rg5 reduced kidney damage by inhibiting the activation of NF-κB/NLRP3 signaling pathway in the HT/STZ diabetic mouse model (217). Huangkui capsule attenuated tubular epithelial-mesenchymal transition in diabetic nephropathy mice by inhibiting TLR4/NF-κB/NLRP3 pathway (218). Gypenosides alleviated diabetic myocardial injury by inhibiting ROS/NLRP3 inflammasome activation (184). Notably, Salidroside alleviated NAFLD in HT diet mice by regulating TXNIP/NLRP3 pathway (219). Sulforaphane inhibited the activation of NLRP3 inflammasome against diabetic retinopathy (220). In addition, Jinmaitong also regulated the NLRP3 pathway to improve STZ-induced DPN in rats (165). In the study of Bai et al., a detailed summary of traditional Chinese medicines targeting the NLRP3 inflammasome pathway to interfere with diabetes progression (167).

Some natural substances also delayed the progression of diabetes and its complications by inhibiting the NLRP3 inflammasome-mediated pyroptosis pathway. For example, melatonin alleviated the pyroptosis of endothelial cells, slowed the progression of atherosclerosis, and inhibited the pyroptosis of neurons and cardiomyocytes in a diabetic mouse model by regulating the NLRP3 axis (199, 221, 222). Similarly, H3 relaxin attenuated pyroptosis in DR and inhibited fibrosis and inflammation in cardiomyocytes of diabetic rats (223, 224). In addition, sodium butyrate (Nab) reduced the pyroptosis of glomerular endothelial cells under HG conditions, and miRNA and LncRNA also played an essential role in the pyroptosis of diabetes and its complications. Targeted knockout or inhibition of RNA expression can reduce the activation of NLRP3 inflammation and delay the progression of diabetes (42, 225–227). According to the existing research evidence, pyroptosis mediated by the NLRP3 inflammasome was commonly found in T2D and its complications. The use of drugs or NLRP3 inflammasome inhibitor MCC950 (196), or even knockout of the NLRP3 gene to inhibit pyroptosis seem to have some effect on T2D Treatment (**Figure 3**) (177). Therefore, targeting the NLRP3 inflammasome may lead to more options for the treatment of diabetic patients in the future.

## Summary and outlook

As a complex metabolic disease, the pathogenesis and complications of diabetes continue to progress. Therefore, the exploration of new treatment modalities and intervention mechanisms is necessary. As a new form of programmed cell death, pyroptosis plays a vital role in the occurrence and development of many diseases. Numerous studies have shown that intervening in the NLRP3 inflammasome signaling pathway can attenuate pyroptosis under HG conditions and delay the progression of complications in animal models of T2D. Targeted regulation of pyroptosis mediated by the NLRP3 inflammasome appears to play a critical role in the progression of diabetes. However, there are still some problems that need to be solved. For example, current studies are all animal models or cell experiments, which are not enough to support targeting the NLRP3 inflammasome to prevent and treat T2D; in addition, the proportion of pyroptosis mediated by the NLRP3 inflammasome in the progression of diabetes is yet unknown, and the research on intervening the mechanism of pyroptosis to delay the progression of T2D is still challenging. However, it is undeniable that the targeted regulation of NLRP3 inflammasome activation has some effect in the treatment of T2D, and some drugs have gradually shown anti-pyroptotic effects. Therefore, an in-depth study of the mechanism of pyroptosis mediated by NLRP3 inflammasome and potential anti-pyroptotic agents may bring some new strategies for treating T2D and drug development.

## Author contributions

XL wrote the manuscript. G-YX and Y-JS participated in literature collection and producing the figures. TG participated in literature collection and edited the manuscript. Q-ML edited the manuscript. XL and Q-ML conceived the study. All authors contributed to the article and approved the submitted version.

## Conflict of interest

The authors declare that the research was conducted in the absence of any commercial or financial relationships that could be construed as a potential conflict of interest.

## Publisher’s note

All claims expressed in this article are solely those of the authors and do not necessarily represent those of their affiliated organizations, or those of the publisher, the editors and the reviewers. Any product that may be evaluated in this article, or claim that may be made by its manufacturer, is not guaranteed or endorsed by the publisher.
